# Hyperchlorhydria, Including a Further Report on the Writer’s Effervescence Test

**Published:** 1899-06

**Authors:** A. L. Benedict

**Affiliations:** Buffalo, N. Y.


### HYPERCHLORHYDRIA, INCLUDING A FURTHER REPORT ON THE WRITER'S EFFERVESCENCE TEST.

A. L. BENEDICT, M. D., Buffalo, N. Y.

SEVERAL years ago, when gastro-enterology depended for diagnosis and treatment very largely on siphonage of the stomach, the writer devoted considerable attention to the problems of studying and medicating the stomach without using the tube. While neither condemning local interference with the stomach nor hesitating to resort to it freely, the writer held that the tube was introduced too often and too indiscriminately, that we failed properly to apply the experience derived from one case to the next, and that patients who objected to the use of the tube from lack of leisure, from exhaustion

1. Read by title at the thirty-first annual meeting of the Medical Association of Central New York, at Auburn, October 18, 1898.

following its use or even from nervousness or actual cowardice regarding its insertion, ought, as a matter of humanity, to have their cases as thoroughly diagnosticated and as well treated as possible.

In the great majority of gastric cases, we have to deal with abnormalities of secretion and motion that are practically functional, though it is quite probable that most of them have a distinct morbid anatomy, if we could only discover it. When we consider that the ferments, pepsin and rennet, are rarely deficient and practically incapable of being present in excess, it is evident that the principal secretory disturbance affects the acidity. Even in essentially motor cases, no direct disturbance of digestion and nutrition occurs till acidity becomes abnormal. In short it may be said that functional dyspepsia, limited to one particular function, causes little disturbance *in esse*, though a very serious matter *in posse*.

It became evident very early that, in any miscellaneous selection of cases the question as to the acidity was by far the most important and the desirability of being able to settle this question, even approximately by external methods, became manifest. The writer's method, first reported several years ago, is extremely simple in theory, though requiring considerable practice before positive deductions can be drawn. It depends upon the production of audible effervescence when a saturated solution of sodium bicarbonate is mixed with an acid solution. Any one who has doubts as to the possibility of producing audible effervescence within the stomach, need only pour a little saturated solution of sodium bicarbonate into a glass containing a 2:1000 solution of hydrochloric acid, the normal maximum or physiologic standard of gastric juice. He will find that the effervescence can be detected at a distance of several inches from the ear. In practice, the stethoscope is placed about two inches north-east from the umbilicus or at the exact level of the stomach contents, as determined by percussion. A few seconds, normally about ten, after the elevation of the larynx indicates that deglutition has begun, a squirting sound is heard, due to the passage of the liquid through the cardia. Care must be taken that the patient does not rustle the clothing, breathe noisily or in other ways interfere with the delicacy of the test. If the stomach contents are decidedly acid, a fizzing sound is heard, which lasts a few seconds. Practice is necessary to distinguish this effervesence from fine borborygmi, occurring as the result of peristaltic waves which the ingestion of food or liquid is apt to produce. Sometimes not even a wave is produced and almost absolute stillness prevails. In this case, to make assurance of nonacidity doubly sure, a glassful of diluted hydrochloric acid, of about normal gastric strength, may be given to the patient, when effervescence will be produced. Of course, the acid drink must be given promptly.

Obviously, the method proposed does not distinguish different kinds of acidity nor is it quantitative. It should, of course, be employed at suitable times after meals, which correspond somewhat to the test-breakfast and the test-dinner, but need not be subject to rigid rules. It is possible to make a distinction between (1) marked subacidity or nonacidity; (2) acidity nearly normal; (3) marked superacidity. In the first case, provided the test is made at the height of digestion and the meal is moderate, there is usually an indication for hydrochloric acid. In the second case, a decision must be made between practically normal secretion of hydrochloric acid and the formation of acids of fermentation in the absence of hydrochloric acid. While the hydrochloric and organic acids may coexist, it is practically impossible to have a serious formation of the latter in a stomach which regularly secretes enough hydrochloric acid. It is rarely that normal effervescence, due to hydrochloric acid, is found in a person presenting himself as a patient and when this does occur, the trouble is almost always supermotility or else a nervous or hysteric dyspepsia. Occasionally, organic stricture at the pylorus coexists with euchlorhydria. When normal effervescence is due to organic acids, there is also formed an abundance of gas which can be detected by percussion, auscultation, sometimes by palpation and by the history of belching gas, which must be sharply discriminated from the account of raising small quantities of sour, watery stomach contents. Practically all the laity and a good many physicians fail to realise that perfectly normal chyme is intensely sour. In the third case, when effervescence is increased and protracted, especially when it occurs with an immediate repetition of the soda test, the diagnosis of hyperchlorhydria may usually be made without hesitation. Theoretically, such a degree of effervescence might be produced by organic acids, acid salts, etc., but even then, other evidences of malfermentation would be conspicuous. An important symptom of hyperchlorhydria, is burning pain in the epigastrium, occurring at the height of digestion, relieved by taking water or food and unaccompanied by gas formation. In three cases seen rather recently, this symptom with increased effervescence was so typic, that no chemic investigation of the stomach contents was made, all the patients recovering under treatment directed against the hyperchlorhydria. A detailed description would be uninteresting, as no corroborative proof of diagnosis can be offered.

The state of the stomach is by no means constant in the same individual. One of the writer's patients who had a rather obstinate subacid dyspepsia—verified by chemic examination, as well as by the lack of effervescence--later developed a typic acute peptic ulcer, which we usually associate with increased acidity as an etiologic factor. Whether acute peptic ulcer is so closely associated with hyperchlorhydria as has been taught, is not absolutely proved. Once formed, the acidity may be increased, deficient or normal and ulcers of the stomach, not of the rodent type, certainly are not directly dependent on hyperchlorhydria. Still it is probably correct to assume that in hyperchlorhydria, we are threatened with ulcer and that acute peptic ulcer rarely develops without hyperchlorhydria.

The effervescence test is of especial value in keeping approximate watch of the course of a case that is occasionally investigated chemically, but it frequently is positive enough to afford a practical basis for treatment without siphonge. The following case illustrates a rather unusual condition:

F. K., aged 35, was treated two years and a half ago for tonic dilatation of the stomach with persistent subacidity, according to the lack of effervescence. February 20th he again presented himself, complaining of burning pain in the epigastrium with sour eructations, but no gas, occurring three or four hours after eating. The stomach was normal in position; effervescence, one hour after light meal, normal. The next day, the stomach contents were obtained one hour after the test meal of 50 grams bread, 5 grams butter, 250 c.c. water. The total acidity by phenolphthalein was 60 per cent., there was no free hydrochloric acid nor more than the normal trace of lactic acid. A slight hemorrhage had occurred and had probably neutralised a small amount of free hydrochloric acid. This case was probably essentially motor, at any rate it was soon relieved without treatment directed toward secretion.

J. L. J., from Pennsylvania, aged 34, gave a typic history of hyperchlorhydria and effervescence was marked and prolonged. As he could spend only a few days in Buffalo, it was thought best to make a chemic examination. On three successive days, his total acidity was 90, 89, 148 per cent.; free HCl being 14, 23 and 35 parts in 10,000, respectively. The first examination did not show absolutely an excess of HCl, but the combined HCl was large.

In another case repeated examinations showed a rather high free hydrochloric acidity, 20, 22, 23, 19 parts in 10,000, but scarcely such as to warrant the term hyperchlorhydria, unless we accept the low standard adopted by Dr. Boardman Reed, of Philadelphia, and others. Yet this case was essentially one of excess of hydrochloric secretion since, one hour after the test meal, the stomaeh contents measured twice the quantity that had been introduced with the meal. In this case the effervescence was always abnormally marked till treatment began to diminish both the bulk and the proportionate acidity of the chyme.

Not to weary you with other illustrative cases, a few words regarding treatment may not be out of order. Gastric ulcer should always be borne in mind as a possible outcome, just as eclampsia in pregnant women with nephritis, but it need not be expected actually to develop in more than a few per cent. of all cases of hyperchlorhydria. How shall the acidity be reduced? Not by alkalies, except when the patient is under the physician's close observation and when the immediate presence of superacidity is demonstrated. The diet should be such as to fix the secreted hydrochloric acid, that is, should be largely proteid, but not such as to stimulate increased secretion: that is, meats and salts should be avoided. Milk, eggs and bread stuffs should constitute the greater part of the diet of such patients. Whenever painful attacks occur, the patient should drink a glassful of cold water, which not only dilutes the acid already represent, but checks the tendency to secretion. If the bowels are constipated an alkaline sulphate or phosphate mixture may be used before breakfast and, perhaps, before the other meals. Gaston Lyon, in the *Bulletin Medicale*, 1898, advises the following formula:

| | | |
|---|---|---|
| ℞ | $NaHCO_3$ . . . . . . . . . . . . . . . . . . . | 40 |
| | $Na_2SO_4$ . . . . . . . . . . . . . . . . . . . . | 40 |
| | NaCl . . . . . . . . . . . . . . . . . . . . . . | 20 |

Sig. Dessertspoonful before breakfast in hot water.

Atropine, cannabis indica and cocaine are of benefit not only as analgesics, but to prevent secretion.

The main point in treatment is overlooked by Lyon. The radical cure consists in checking the tendency to formation of too much HCl; the temporary cure and that which leads up to the radical cure, consists in preventing the actual secretion of an excessive amount of HCl. This secretion obviously depends on the presence of chlorides, of which NaCl is the most abundant and the most important. Salt should be absolutely tabooed, though it is not necessary to administer food and water chemically free from saline ingredients. If no salt is allowed either in the kitchen or on the table and if water is drunk freely, the tissues and fluids soon become unable to surrender any chlorine to the gastric oxyntic cells and the hyperchlorhydria of necessity abates.

174 FRANKLIN STREET.

